# Inverse Thermoelastic Analysis of a Cylindrical Tribo-Couple

**DOI:** 10.3390/ma14102657

**Published:** 2021-05-19

**Authors:** Roman Kushnir, Anatoliy Yasinskyy, Yuriy Tokovyy, Eteri Hart

**Affiliations:** 1 Pidstryhach Institute for Applied Problems of Mechanics and Mathematics, National Academy of Sciences of Ukraine, 79060 Lviv, Ukraine; dyrector@iapmm.lviv.ua (R.K.); yasinskyy.anatoliy@gmail.com (A.Y.); 2 Department of Applied Mathematics, Institute of Applied Mathematics and Fundamental Sciences, National University Lviv Polytechnic, 79013 Lviv, Ukraine; 3 Department of Theoretical and Computer Mechanics, Oles Honchar Dnipro National University, 49010 Dnipro, Ukraine; hart@ua.fm

**Keywords:** tribo-couple, cylindrical layers, frictional heating, unknown thermal loading, inverse thermoelasticity problem, Volterra integral equation, stable algorithm

## Abstract

Within the framework of the one-dimensional model for a tribo-couple consisting of two elastic cylinders accounting for the frictional heat generation on the interface due to the roughness of the contacting dissimilar materials, a problem on the identification of the unknown temperature on one of the limiting surfaces of either inner or outer cylindrical layers is formulated and reduced to an inverse thermoelasticity problem via the use of the circumferential strain given on the other surface. To solve the latter problem, a semi-analytical algorithm is suggested, and its stability with respect to the small errors in the input data is analyzed. The efficiency of the proposed solution algorithm is validated numerically by comparing its results with the solution of a corresponding direct problem. The temperature and thermal stresses in the tribo-couple are analyzed.

## 1. Introduction

Construction and improvement of the elements of present-day techniques, along with the development and implementation of new materials with advanced properties, necessitate the comprehensive analysis of the heat transfer and the stress–strain state in composite materials under the simultaneous action of force and thermal fields while accounting for a wide range of the operational and constructional features, as well as the interaction of the structural elements of different geometry [1]. The importance of such analysis for both mechanical engineering and material science is also motivated by the prioritized implementation of non-destructive testing, which is important for ensuring the safety and durability of the operational performance of the heat and power equipment [2,3,4].

The comprehensive thermoelastic analysis is extremely important for the structural elements, some surface parts of which appear to be inaccessible (due to specific structural, technological, operational, or environmental reasons) for the direct reading of the thermal and mechanical signatures that are to be in use as the boundary conditions for the corresponding direct heat-transfer and thermoelasticity problems. As a result, the corresponding heat-transfer and thermoelasticity problems for such structural elements become ill-posed and require some supplementary information about the thermal or mechanical process, collected, preferably, on the accessible segments of the surface. It is worth noting that the type of additional information can be regarded as a critical point of the methodologies for solving the ill-posed problems of this kind. 

If, for example, the original problem is supplemented with the information about some parameters of the thermal process (e.g., temperature or heat flux) at some points of a solid or its surface, the problem of the identification of the unknown thermal loading can be reduced to solving an inverse heat conduction problem [5,6]. The inverse problems obtained in this case are substantially ill-posed and their solution is concerned with the application of the corresponding regularizing algorithms.

There are numerous practical cases, however, when the reproduction of all the components of thermal loading appears to be impossible within the framework of an inverse heat-transfer problem alone. While accounting for coupling between the temperature and strain fields, the original mathematical models in these cases can be extended to non-classical thermoelastic problems by implementing the additional information on the thermomechanical parameters (displacements, strains, or stresses) on the accessible segment of the surface. The problem of determining the temperature and thermostressed fields in a solid under the above conditions can thereby be reduced to an inverse thermoelasticity problem [7]. The inverse problems of this kind are conditionally well-posed; i.e., they may become well-posed under certain supplementary conditions. This can be explained by the fact that the components of the stress–strain state have the form of integral dependences on the temperature at all points of a solid, including its boundary [1,8,9]. For non-stationary processes, these conditions usually express the fitting between the input data at the initial moment of time or interrelation of the mechanical components on the surfaces of the solid [1,10]. Some methods for solving one- and two-dimensional inverse thermoelasticity problems have been addressed in [11,12,13,14,15,16]. 

The inverse analysis of the temperature on inaccessible surfaces is an important issue in the analysis of tribo-systems. Such analysis is a key point in evaluating the characteristics of frictional interaction and material properties and is vital for both mechanical engineering and material science. Therefore, many practical cases of thermoelasticity problems, those that focus on the coupling between the thermal and mechanical fields, are concerned with frictional heating induced by the roughness of the contacting surfaces of interacting solids (see, e.g., [17,18,19]).

In [20,21,22], a technique for solving the inverse thermoelasticity problems was presented based on the reduction to integral equations. Particularly in [21,22], one-dimensional thermoelasticity problems were considered for interacting layers with friction. In engineering practice and material science experiments, numerous tribo-systems involve elements of cylindrical shape. In this paper, we extend the technique for solving inverse one-dimensional thermoelasticity problems, which are obtained for the identification of the time-dependence of a temperature of one of the circumferences of a cylindrical tribo-couple by making use of the additionally known circumferential strain on the surface where the thermal loading is known.

## 2. Formulation of the Problem

**Figure 1:**
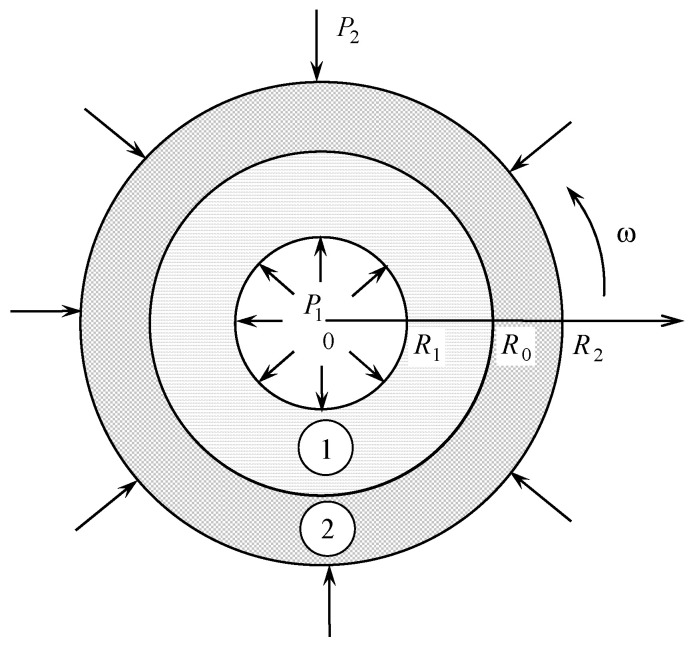
The scheme of the considered tribo-couple, where the inner and outer cylindrical layers are denoted by “1” and “2”, respectively; the thermal and force loadings are imposed on the inner and outer surfaces $R_{1}$ and $R_{2}$ and the frictional heating occurs on the interface $R_{0}$.

Consider a one-dimensional model of a cylindrical tribo-couple consisting of two cylindrical elements made of dissimilar materials generating heat due to mutual interfacial friction [17]. The model is schematized in Figure 1 and presented by a circular hollow cylinder “1” of the inner and outer radii $r=R_{1}$ and $r=R_{0}$ encapsulated without tension and gap into another cylinder “2” of the same shape with inner and outer radii $r=R_{0}$ and $r=R_{2}$, respectively. Assume the inner, $r=R_{1}$, and outer, $r=R_{2}$, circumferences of the tribo-couple to be kept under the given transient temperatures $t_{1}^{*}(\tau_{*})$ and $t_{2}^{*}(\tau_{*})$ while being subjected to the compressive forces $P_{1}(\tau_{*})$ and $P_{2}(\tau_{*})$. Here, r is the radial coordinate and $\tau_{*}$ is time. The mechanical and thermal contact of the cylindrical layers occurring on the interface $r=R_{0}$ is assumed to be imperfect in view of the roughness of the material on contacting surfaces so that the linear relationship

(1) $${\tilde{u}}^{\left(i\right)}(\tau_{*})={\left(-1\right)}^{i}n_{i}P(\tau_{*}),\;i=1,2$$

obtains between the radial displacements on the interface ${\tilde{u}}^{\left(1\right)}(\tau_{*})$ and ${\tilde{u}}^{\left(2\right)}(\tau_{*})$ induced by the deformation of micro-roughness and the contact pressure $P(\tau_{*})$. Here, $n_{i}$ are the coefficients characterizing the deformative features of the contacting surfaces.

Assume that one of the cylinders (let it be the outer one) rotates against the other cylinder. Let us restrict our attention to the case when the rotation reaches a steady-state condition at a constant angular velocity ω=const. Due to the frictional forces according to Amonton’s law, the interface $r=R_{0}$ is subjected to the non-stationary heat generation, and the specific power of the frictional heating sources equals the specific work of the friction forces. The mechanical and thermo-physical properties of cylinders 1 and 2 are constant and indicated with upper indices accordingly. Within the framework of the formulated problem, the transient temperature field in the considered tribo-couple varies along the radial coordinate r only, and in view of the plane strain condition, $u_{z}^{\left(1\right)}=u_{z}^{\left(2\right)}=0$, where $u_{z}^{\left(i\right)}$ is the axial displacement of the ith layer of the cylinder.

In view of the foregoing model, the one-dimensional thermoelasticity problem for the tribo-couple is governed by the following system of equations, including:

(*i*) the heat-transfer equation

(2) $$\frac{\partial^{2}T_{i}^{*}(\rho ,\tau )}{\partial \rho^{2}}+\frac{1}{\rho }\frac{\partial T_{i}^{*}(\rho ,\tau )}{\partial \rho }=\frac{1}{b_{i}}\frac{\partial T_{i}^{*}(\rho ,\tau )}{\partial \tau }$$

(*ii*) and the Lamé equations

(3) $$\frac{\partial^{2}u_{r}^{\left(i\right)}(\rho ,\tau )}{\partial \rho^{2}}+\frac{1}{\rho }\frac{\partial u_{r}^{\left(i\right)}(\rho ,\tau )}{\partial \;\rho }-\frac{u_{r}^{\left(i\right)}(\rho ,\tau )}{\rho^{2}}=\beta_{i}R_{0}\frac{\partial \;T_{i}^{*}(\rho ,\tau )}{\partial \rho }$$

(4) $$\frac{\partial^{2}u_{\varphi }^{\left(i\right)}(\rho ,\tau )}{\partial \rho^{2}}+\frac{1}{\rho }\frac{\partial u_{\varphi }^{\left(i\right)}(\rho ,\tau )}{\partial \rho }-\frac{u_{\varphi }^{\left(i\right)}(\rho ,\tau )}{\rho^{2}}=0$$

under the set of complementary conditions consisting of:

(*i*) the mechanical boundary conditions

(5) $$\sigma_{rr}^{\left(i\right)}(k_{i},\tau )=-P_{i}(\tau ),\;u_{\varphi }^{\left(i\right)}(k_{i},\tau )=0$$

(*ii*) the mechanical interface conditions

(6) $$\begin{array}{c} \sigma_{rr}^{\left(1\right)}(1,\tau )=\sigma_{rr}^{\left(2\right)}(1,\tau )=-P(\tau ),\;\sigma_{r\varphi }^{\left(1\right)}(1,\tau )=\sigma_{r\varphi }^{\left(2\right)}(1,\tau )=-fP(\tau )\\ u_{r}^{\left(1\right)}(1,\tau )+{\tilde{u}}^{\left(1\right)}(\tau )=u_{r}^{\left(2\right)}(1,\tau )+{\tilde{u}}^{\left(2\right)}(\tau ),\;u_{z}^{\left(i\right)}(\rho ,\tau )=0 \end{array}$$

(*iii*) the thermal boundary conditions

(7) $$T_{i}^{*}(k_{i},\tau )=t_{i}^{*}(\tau )$$

(*iv*) the thermal interface conditions

(8) $$\begin{array}{c} \lambda_{1}\frac{\partial T_{1}^{*}(1,\tau )}{\partial \;\rho }-\lambda_{2}\frac{\partial \;T_{2}^{*}(1,\tau )}{\partial \;\rho }=\omega R_{0}^{2}fP(\tau )\\ \lambda_{1}\frac{\partial \;T_{1}^{*}(1,\tau )}{\partial \rho }+\lambda_{2}\frac{\partial \;T_{2}^{*}(1,\tau )}{\partial \rho }=\frac{R_{0}}{R}(T_{2}^{*}(1,\tau )-T_{1}^{*}(1,\tau )) \end{array}$$

(*v*) and the initial condition

(9) $$\;T_{i}^{*}(\rho ,0)=T_{0}=\mathrm{const}\neq 0$$

where, i=1,2 (i=1 corresponds to the range $\rho \in [k_{1},1)$ and i=2 corresponds to the range $\rho \in (1,k_{2}])$, $\rho =r/R_{0}$ is the dimensionless radial coordinate $\rho \in [k_{1},k_{2}]$, $k_{i}=R_{i}\;/\;R_{0}$, $\tau =a_{2}\tau_{*}/R_{0}^{2}$ is the Fourier criterion, $\tau \in (0,\tau_{m}]$, $\tau_{m}$ is a constant parameter, $b{\;}_{1}=a_{1}\;/a_{2}$, $b_{2}=1$, $a{\;}_{i}$ is the coefficient of thermal diffusivity, $\beta {\;}_{i}=\alpha_{T}^{\left(i\right)}(1+\nu_{i})/(1-\nu_{i})$, $\alpha_{T}^{\left(i\right)}$ is the coefficient of linear thermal expansion, $\nu_{i}$ is the Poisson ratio, $\lambda_{i}$ denotes the heat-conduction coefficient, $T_{i}^{*}$ is the temperature, $u_{r}^{\left(i\right)}$ and $u_{\varphi }^{\left(i\right)}$ are the radial and circumferential displacements, $\sigma_{rr}^{\left(i\right)}$ and $\sigma_{r\varphi }^{\left(i\right)}$ are the radial and tangential stress-tensor components, f is the coefficient of friction, and R is the coefficient of contact thermal resistance.

It is well known that in the case of plane strain, the thermoelasticity problem (3) and (4) can be represented by two independent problems [23], when (i) $u_{r}^{\left(i\right)}\neq 0$, $\varepsilon_{rr}^{\left(i\right)}\neq 0$, $\varepsilon_{\varphi \varphi }^{\left(i\right)}\neq 0$, $u_{\varphi }^{\left(i\right)}=$ $\varepsilon_{r\varphi }^{\left(i\right)}=0$ and (ii) $u_{\varphi }^{\left(i\right)}\neq 0$, $\varepsilon_{r\varphi }^{\left(i\right)}\neq 0$, $u_{r}^{\left(i\right)}=$$\varepsilon_{rr}^{\left(i\right)}=$$\varepsilon_{\varphi \varphi }^{\left(i\right)}=0$, i=1,2. Here, $\varepsilon_{rr}^{\left(i\right)},$ $\varepsilon_{\varphi \;\varphi }^{\left(i\right)}$, and $\varepsilon_{r\varphi }^{\left(i\right)}$ are, respectively, the radial, circumferential, and tangential strains of the ith cylindrical layer.

If all the input functions and coefficients in Equations (2)–(9) are properly imposed, then the formulated problem appears to be a well-posed direct thermoelasticity problem. Assuming, however, the transient temperature $t_{1}^{*}\left(\tau \right)$, $\tau \in [0,\;\tau_{m}]$, on the inner surface $\rho =k_{1}$ to be unknown (a typical situation due to the inaccessibility of the inner surface for the direct measurement) necessitates the determination of this function prior to solving the direct problem.

In order to identify this function appearing in the boundary condition (7), we use the supplementary information about the thermo-mechanical state of the compound cylinder, i.e., the condition

(10) $$\;\varepsilon_{\varphi \varphi }^{\left(2\right)}(k_{2},\tau )=\varphi_{*}(\tau ),\;\tau \in [0,\;\tau_{m}]$$

imposing the circumferential strain measured on the accessible outer surface $\rho =k_{2}$. Here, $\varphi_{*}(\tau )$ is a given function of time.

Let us determine the temperature field and thermal stresses in the considered tribo-couple by making use of condition (10) in order to identify the unknown temperature distribution $t_{1}^{*}\left(\tau \right)$ on the inner circumference of the cylinder.

## 3. Solution Technique

By implementing the technique suggested in [17], a solution to the formulated thermoelastic problem (1), (3)–(6) can be given in the form expressing the circumferential strain in the cylindrical tribo-couple explicitly through the force loadings and thermal field as follows:

(11) $$\varepsilon_{\varphi \varphi }^{\left(i\right)}(\rho ,\tau )=\alpha_{T}^{\left(i\right)}(1+\nu_{i})T_{0}+\left((1-{\bar{\nu }}_{i})+\frac{1+{\bar{\nu }}_{i}}{\rho^{2}}\right)\frac{c_{i}p_{i}(\tau )}{2}\;\;-\left((1-{\bar{\nu }}_{i})+k_{i}^{2}\frac{1+{\bar{\nu }}_{i}}{\rho^{2}}\right)\frac{c_{i}p(\tau )}{2k_{i}^{2}}+{\left(-1\right)}^{i+1}\frac{1-{\bar{\nu }}_{i}}{1+{\bar{\nu }}_{i}}\frac{\beta_{i}T_{0}}{1-k_{i}^{2}}\overset{v_{2}^{\left(i\right)}}{\underset{v_{1}^{\left(i\right)}}{\int }}\xi T_{i}(\xi ,\tau )d\xi \;+\frac{\beta_{i}T_{0}}{2\rho^{2}}\overset{v_{2}^{\left(i\right)}}{\underset{v_{1}^{\left(i\right)}}{\int }}\xi \left({\left(-1\right)}^{i+1}\frac{1+k_{i}^{2}}{1-k_{i}^{2}}+\mathrm{sgn}(\rho -\xi )\right)T_{i}(\xi ,\tau )d\xi$$

and

(12) $$c_{3}p(\tau )=c_{1}p_{1}(\tau )-c_{2}p_{2}(\tau )+\ell_{1}\overset{1}{\underset{k_{1}}{\int }}\xi T_{1}(\xi ,\tau )d\xi +\ell_{2}\overset{k_{2}}{\underset{1}{\int }}\xi T_{2}(\xi ,\tau )d\xi +({\bar{\alpha }}_{1}-{\bar{\alpha }}_{2})T_{0}$$

where i=1,2, $\;p(\tau )=P(\tau )/\sigma_{*}$ and $\;p_{i}(\tau )=P_{i}(\tau )/\sigma_{*}$ are the dimensionless contact pressure and compressive pressures on the inner and outer surfaces, $\sigma_{*}$ is a constant in the dimension of stresses, ${\bar{\nu }}_{i}=\nu_{i}/(1-\nu_{i})$, $\;{\bar{E}}_{i}=E_{i}/(1-\nu_{i}^{2})$, $\;T_{i}=(T_{i}^{*}-T_{0})/T_{0}$, $\;v_{i}^{\left(i\right)}=k_{i}$, $\;v_{1}^{\left(2\right)}=v_{2}^{\left(1\right)}=1$, $\ell_{i}=2\alpha_{T}^{\left(i\right)}(1+\nu_{i})T_{0}/(1-k_{i}^{2})$, $\;{\bar{\alpha }}_{i}=\alpha_{T}^{\left(i\right)}(1+\nu_{i})$, $c_{i}=2k_{i}^{2}\sigma_{*}/((1-k_{i}^{2}){\bar{E}}_{i})$, $E_{i}$ denotes the Young modulus of the *i*th cylindrical layer, and

$$c_{3}=\sum_{i=1}^{2}{\left(-1\right)}^{i+1}\frac{1-{\bar{\nu }}_{i}+(1+{\bar{\nu }}_{i})k_{i}^{2}}{1-k_{i}^{2}}\frac{\sigma_{*}}{{\bar{E}}_{i}}+\frac{(n_{1}+n_{2})\sigma_{*}}{R_{0}}$$

A general solution to Equation (4) for the circumferential strain $u_{\varphi }^{\left(i\right)}$ can be given [23] as

$$u_{\varphi }^{\left(i\right)}(\rho ,\tau )=\frac{A{\;}_{i}(\tau )R_{0}\rho }{2}+\frac{B_{i}(\tau )}{R_{0}\rho }$$

where $A{\;}_{i}\;(\tau )$ and $B{\;}_{i}(\tau )$ are arbitrary and yet unknown functions of time, i=1,2. By making use of conditions (5) and (6) for the displacement $u_{\varphi }^{\left(i\right)}$ and stress $\;\sigma_{r\varphi }^{\left(i\right)}$, we can finally derive

(13) $$u_{\varphi }^{\left(i\right)}(\rho ,\tau )=\frac{f(1+{\bar{\nu }}_{i})R_{0}P(\tau )}{{\bar{E}}_{i}\rho }\left(1-\frac{\rho^{2}}{k_{i}^{2}}\right)\;\varepsilon_{r\varphi }^{\left(i\right)}(\rho ,\tau )=-\frac{f(1+{\bar{\nu }}_{i})P(\tau )}{{\bar{E}}_{i}\rho^{2}},\;\sigma_{r\;\varphi }^{\left(i\right)}(\rho ,\tau )=-\frac{fP(\tau )}{\rho^{2}}$$

Equation (13) allow for expressing the thermal stresses and displacements in the two-layer cylindrical tribo-couple through the contact pressure found by formula (12).

Assuming the function $t_{1}^{*}(\tau )$ to be known for τ≥0 and making use of the integral Laplace transform [24] by the time-variable τ yields a solution to the heat-conduction problem (2), (7), (8) and (9) in the form as follows

(14) $$T_{i}(\rho ,\tau )=\sum_{j=1}^{2}\overset{\tau }{\underset{0}{\int }}G_{j}^{\left(i\right)}(\rho ,\tau -\xi )t_{j}(\xi )d\xi +\Omega \overset{\tau }{\underset{0}{\int }}G_{3}^{\left(i\right)}(\rho ,\tau -\xi )p(\xi )d\xi$$

where i=1,2,

$$G_{1}^{\left(1\right)}(\rho ,\tau )=\sum_{n=1}^{\infty }\frac{\exp(-\mu_{n}^{2}\tau )}{\partial_{s}(\Delta (s_{n}))}(2\lambda Z_{10}^{\left(1\right)}(1,\rho ,s_{n})\;Z_{10}^{\left(2\right)}(1,k_{2},s_{n})\;+\vartheta (\lambda Z_{00}^{\left(2\right)}(k_{2},1,s_{n})Z_{10}^{\left(1\right)}(1,\rho ,s_{n})+Z_{10}^{\left(2\right)}(1,k_{2},s_{n})Z_{00}^{\left(1\right)}(1,\rho ,s_{n})\;)\;)\;G_{2}^{\left(1\right)}(\rho ,\tau )=\vartheta \sum_{n=1}^{\infty }\frac{\exp(-\mu_{n}^{2}\tau )}{\partial_{s}(\Delta (s_{n}))}\;Z_{00}^{\left(1\right)}(\rho ,k_{1},s_{n})\;G_{3}^{\left(1\right)}(\rho ,\tau )=\sum_{n=1}^{\infty }\frac{\exp(-\mu_{n}^{2}\tau )}{\partial_{s}(\Delta (s_{n}))}\;\left(Z_{10}^{\left(2\right)}(1,k_{2},s_{n})+\vartheta Z_{00}^{\left(2\right)}(k_{2},1,s_{n})\right)\;Z_{00}^{\left(1\right)}(\rho ,k_{1},s_{n})\;G_{1}^{\left(2\right)}(\rho ,\tau )=-\lambda \vartheta \sum_{n=1}^{\infty }\frac{\exp(-\mu_{n}^{2}\tau )}{\partial_{s}(\Delta (s_{n}))}\;Z_{00}^{\left(2\right)}(\rho ,k_{2},s_{n})\;G_{2}^{\left(2\right)}(\rho ,\tau )=\sum_{n=1}^{\infty }\frac{\exp(-\mu_{n}^{2}\tau )}{\partial_{s}(\Delta (s_{n}))}(2\lambda Z_{10}^{\left(1\right)}(1,k_{1},s_{n})\;Z_{10}^{\left(2\right)}(1,\rho ,s_{n})\;+\vartheta (\lambda Z_{10}^{\left(1\right)}(1,k_{1},s_{n})Z_{00}^{\left(2\right)}(\rho ,1,s_{n})+Z_{00}^{\left(1\right)}(1,k_{1},s_{n})Z_{10}^{\left(2\right)}(1,\rho ,s_{n})))\;G_{3}^{\left(2\right)}(\rho ,\tau )=-\sum_{n=1}^{\infty }\frac{\exp(-\mu_{n}^{2}\tau )}{\partial_{s}(\Delta (s_{n}))}\;(\lambda Z_{10}^{\left(1\right)}(1,k_{1},s_{n})+\vartheta Z_{00}^{\left(1\right)}(1,k_{1},s_{n}))\;Z_{00}^{\left(2\right)}(\rho ,k_{2},s_{n})\;\Delta (s)=2\lambda Z_{10}^{\left(2\right)}(1,k_{2},s)Z_{10}^{\left(1\right)}(1,k_{1},s)\;\;+\vartheta \left(\lambda Z_{10}^{\left(1\right)}(1,k_{1},s)Z_{00}^{\left(2\right)}(k_{2},1,s)+Z_{10}^{\left(2\right)}(1,k_{2},s)Z_{00}^{\left(1\right)}(1,k_{1},s)\right)\;Z_{10}^{\left(j\right)}(x,y,s)=q_{j}x(I_{1}(q_{j}x)K_{0}(q_{j}y)+I_{0}(q_{j}y)K_{1}(q_{j}x))\;Z_{kk}^{\left(j\right)}(x,y,s)=I_{k}(q_{j}x)K_{k}(q_{j}y)+I_{k}(q_{j}y)K_{k}(q_{j}x)$$

j=1,2; k=0,1, $\Omega =\omega R_{0}^{2}f\sigma_{*}/(\lambda_{2}T_{0})$ is the dimensionless angular velocity, $\lambda =\lambda_{1}/\lambda_{2}$, $\vartheta =R_{s}/\bar{R}$, $R_{s}=R_{0}/(R_{*}\lambda_{2})$, $q_{1}^{2}=s/b_{1}$, $q_{2}^{2}=s$, $\bar{R}=R/R_{*}$ is the dimensionless interfacial thermal resistance, $R_{*}$ is a constant in the dimension of thermal resistance, $I_{k}(s)$ and $K_{k}(s)$ are the modified Bessel functions of the first and second kind, k=0,1, s stands for the parameter of the Laplace transform, $\partial_{s}$ denotes the partial derivative by s, and $s_{n}=-\mu_{n}^{2}$ are the roots of the characteristic equation Δ(s)=0, $\mu_{n}>0,\;n=1,2,\;\ldots$

Formula (14) expresses the dependence of the temperature field within the tribo-couple on the contact pressure, while formula (12) shows the dependence of the contact pressure on the temperature. By making use of these two formulas along with expression (11) for the circumferential strain, the condition for the radial displacement in (5) and (6) yields the following formula for the contact pressure on the interface:

(15) $$p(\tau )=\overset{\tau }{\underset{0}{\int }}M(\tau -\eta )\left(c_{1}p_{1}(\eta )-c_{2}p_{2}(\eta )\right)d\eta +\sum_{i=1}^{2}\overset{\tau }{\underset{0}{\int }}N_{i}(\tau -\eta )t_{i}(\eta )d\eta \;+\left((1+\nu_{1})\alpha_{T}^{\left(1\right)}-(1+\nu_{2})\alpha_{T}^{\left(2\right)}\right)T_{0}\overset{\tau }{\underset{0}{\int }}M(\eta )d\eta$$

where

$$M(\tau )=\sum_{n=1}^{\infty }\frac{\Delta (s_{n}^{*})\exp(s_{n}^{*}\tau )}{\partial_{s}(\Delta_{*}(s_{n}^{*}))}\;,\;N_{i}(\tau )=\sum_{n=1}^{\infty }\frac{V_{i}(s_{n}^{*})\exp(s_{n}^{*}\tau )}{\partial_{s}(\Delta_{*}(s_{n}^{*}))}\;V_{1}(s)=\ell_{1}(2\lambda k_{1}Z_{11}^{\left(1\right)}(1,k_{1},s)Z_{10}^{\left(2\right)}(1,k_{2},s)+\vartheta (\lambda k_{1}Z_{11}^{\left(1\right)}(1,k_{1},s)Z_{00}^{\left(2\right)}(k_{2},1,s)\;+\;Z_{10}^{\left(2\right)}(1,k_{2},s)\frac{Z_{10}^{\left(1\right)}(k_{1},1,s)-1}{q_{1}^{2}}))-\ell_{2}\lambda \vartheta \frac{1-Z_{10}^{\left(2\right)}(1,k_{2},s)}{q_{2}^{2}}\;V_{2}(s)=\ell_{1}\vartheta \frac{Z_{10}^{\left(1\right)}(1,k_{1},s)-1}{q_{1}^{2}}+\ell_{2}(2\lambda k_{2}Z_{10}^{\left(1\right)}(1,k_{1},s)Z_{11}^{\left(2\right)}(k_{2},1,s)\;+\vartheta (\begin{array}{c} \;\\ \; \end{array}\lambda Z_{10}^{\left(1\right)}(1,k_{1},s)\frac{Z_{10}^{\left(2\right)}(k_{2},1,s)-1}{q_{2}^{2}}+k_{2}Z_{00}^{\left(1\right)}(1,k_{1},s)Z_{11}^{\left(2\right)}(k_{2},1,s)\;\begin{array}{c} \;\\ \; \end{array}))\;\Delta_{*}(s)=c_{3}\Delta (s)-\Omega (\ell_{1}\left(Z_{10}^{\left(2\right)}(1,k_{2},s)+\vartheta Z_{00}^{\left(2\right)}(k_{2},1,s)\right)\frac{Z_{10}^{\left(1\right)}(1,k_{1},s)-1}{q_{1}^{2}}\;-\ell_{2}\left(\lambda Z_{10}^{\left(1\right)}(1,k_{1},s)+\vartheta Z_{00}^{\left(1\right)}(1,k_{1},s)\right)\frac{1-Z_{10}^{\left(2\right)}(1,k_{2},s)}{q_{2}^{2}})$$

$s_{n}^{*}$ are the roots of the characteristic equation $\Delta_{*}(s)=0$, n=1,2, …

By putting (14) and (15) into formula (11) at i=2 and $\rho =k_{2}$ within the context of the supplementary condition (10) for the circumferential strain, we arrive at the convolution-type Volterra integral equation of the first kind [25] for the determination of function $t_{1}(\tau )$ in the following form:

(16) $$\overset{\tau }{\underset{0}{\int }}K_{1}(\tau -\eta )\;t_{1}(\eta )d\eta =\varphi_{*}(\tau )-\overset{\tau }{\underset{0}{\int }}K_{2}(\tau -\eta )\;t_{2}(\eta )d\eta \;-\overset{\tau }{\underset{0}{\int }}L(\tau -\eta )\;\left(c_{1}p_{1}(\eta )-c_{2}p_{2}(\eta )\right)d\eta -\frac{c_{2}}{2k_{2}}(1+k_{2}^{2}+(1-k_{2}^{2}){\bar{\nu }}_{2})p_{2}(\tau )\;-\left((1+\nu_{1})\alpha_{T}^{\left(1\right)}-(1+\nu_{2})\alpha_{T}^{\left(2\right)}\right)T_{0}\overset{\tau }{\underset{0}{\int }}L(\eta )d\eta -(1+\nu_{2})k_{2}\alpha_{T}^{\left(2\right)}T_{0}$$

where $\tau \in [0,\tau_{m}]$ and

$$K_{i}(\tau )=\sum_{k=1}^{\infty }\frac{U_{i}^{}(s_{k})\exp(s_{k}\tau )}{\partial_{s}(\Delta (s_{k})\Delta_{*}(s_{k}))},\;L(\tau )=\sum_{n=1}^{\infty }\frac{V_{3}(s_{n}^{*})\exp(s_{n}^{*}\tau )}{\partial_{s}(\Delta_{*}(s_{n}^{*}))}\;U_{1}(s)=V_{1}(s)V_{3}(s)+k_{2}\ell_{2}\lambda \vartheta \frac{1-Z_{10}^{\left(2\right)}(1,k_{2},s)}{q_{2}^{2}}\Delta_{*}(s)\;U_{2}(s)=V_{2}(s)V_{3}(s)-k_{2}\ell_{2}(2\lambda k_{2}Z_{10}^{\left(1\right)}(1,k_{1},s)Z_{11}^{\left(2\right)}(k_{2},1,s)\;\;+\vartheta (\lambda Z_{10}^{\left(1\right)}(1,k_{1},s)\frac{Z_{10}^{\left(2\right)}(k_{2},1,s)-1}{q_{2}^{2}}+k_{2}Z_{00}^{\left(1\right)}(1,k_{1},s)Z_{11}^{\left(2\right)}(k_{2},1,s)\;))\Delta_{*}(s)\;V_{3}(s)=-\frac{c_{2}}{k_{2}}\Delta (s)+\Omega \;k_{2}\ell_{2}\left(\lambda Z_{10}^{\left(1\right)}(1,k_{1},s)+\vartheta \;Z_{00}^{\left(1\right)}(1,k_{1},s)\right)\frac{1-Z_{10}^{\left(2\right)}(1,k_{2},s)}{q_{2}^{2}}$$

$s_{k}$ are roots of equations Δ(s)=0 and $\Delta_{*}(s)=0$ combined, which are negative real numbers $s_{k}=-\gamma_{k}^{2}$, $\gamma_{k}>0$, k=1,2,…, when the angular velocity does not exceed a critical value [17].

By setting τ=0 in (16) and allowing $t_{i}(0)=0$, i=1,2, we derive the fitting condition for the initial temperature, the circumferential strain imposed on the outer surface $\rho =k_{2}$, and the dimensionless pressures on the inner and outer circumferences of the tribo-couple at the initial moment of time in the form as follows:

$$\frac{c_{2}}{2k_{2}}(1+k_{2}^{2}+(1-k_{2}^{2}){\bar{\nu }}_{2})p_{2}(0)-\frac{c_{2}}{k_{2}c_{3}}(c_{1}p_{1}(0)-c_{2}p_{2}(0))\;-\frac{c_{2}}{k_{2}c_{3}}((1+\nu_{1})\alpha_{T}^{\left(1\right)}-(1+\nu_{2})\alpha_{T}^{\left(2\right)})T_{0}+(1+\nu_{2})k_{2}\alpha_{T}^{\left(2\right)}T_{0}=\varphi_{*}(0)$$

The latter condition ensures the continuity of the solution of integral Equation (16).

In such a manner, the original heat-conduction problem for the considered cylindrical tribo-couple with frictional hating is reduced to an inverse thermoelasticity problem, which is verbalized by the integral Equation (16) and implies the determination of the temperature on the inner surface via the temperature and circumferential strain given on the outer surface.

It can be shown that the kernel $K_{1}(\tau -\eta )$ of Equation (16) is always positive for η∈[0,τ], increases monotonically and suffers the root singularity at η=τ. This means that Equation (16) is the Abel integral equation [25]. The fact that the kernel $K_{1}(\tau -\eta )$ has the integrable singularity at η=τ implies the absence of the time delay in the maximum response of the thermal constituent of the circumferential strain $\varphi_{*}(\tau )$ to the variation of temperature $t_{1}(\tau )$.

Assume the unknown temperature $t_{1}(\eta )$ to be a continuous function on the interval [0,τ], i.e., $t_{1}(\eta )\in C_{\left[0,\tau \right]}$, to construct a solution to Equation (16). Let us represent the time interval $\left[0,\tau_{m}\right]$ by the mesh consisting of m intervals of the length $h=\tau_{m}/m$ and represent the sought-out function on each of these intervals by a linear spline $S_{j}^{\left(1\right)}(\tau )=((\tau_{j}-\tau )t_{1}^{\left(j-1\right)}+(\tau -\tau_{j-1})t_{1}^{\left(j\right)})h^{-1},$ $\tau \in [\tau_{j-1},\tau_{j}],$ $\tau_{j}=hj,$ $t_{1}^{\left(j\right)}=t_{1}(\tau_{j}),$ j=1, … ,m. As a result, Equation (16) yields the following system of linear algebraic equations:

(17) $$t{\;}_{1}^{\left(1\right)}=\frac{\Phi_{1}}{c_{0}},\;\sum_{j=1}^{l-1}\Theta_{l\;j}t{\;}_{1}^{\left(j\right)}+t{\;}_{1}^{\left(l\right)}=\frac{\Phi_{l}}{c_{0}},\;l=2,\ldots ,m$$

Here, $\Phi_{l}=\Phi (\tau_{l})$ is the values of the right-hand side of Equation (16) at the knots of the mesh $\tau =\tau_{l}$ and

$$\Theta_{lj}\equiv q_{l-j}=\frac{1}{c_{0}}\sum_{k=1}^{\infty }\frac{U_{1}(s_{k})}{\partial_{s}(\Delta (s_{k})\Delta_{*}(s_{k}))}\;\frac{{\left(1-\exp(-\gamma_{k}^{2}h)\right)}^{2}}{\gamma_{k}^{4}h^{2}}\exp(-\gamma_{k}^{2}h(l-j-1)),\;j<l\;c{\;}_{0}=\sum_{k=1}^{\infty }\frac{U_{1}(s_{k})}{\partial_{s}(\Delta (s_{k})\Delta_{*}(s_{k}))}\;\frac{1}{\gamma_{k}^{2}h}\left(1-\frac{1-\exp(-\gamma_{k}^{2}h)}{\gamma_{k}^{2}h}\right)$$

The matrix of system (17) is the lower diagonal matrix with equal elements on each diagonal below the main one:

$$Q_{1}=\left(\begin{array}{cccccc} 1 & 0 & 0 & \cdots & 0 & 0\\ q_{1} & 1 & 0 & \cdots & 0 & 0\\ q_{2} & q_{1} & 1 & \cdots & 0 & 0\\ \vdots & \vdots & \vdots & \ddots & \vdots & \vdots \\ q_{m-1} & q_{m-2} & q_{m-3} & \cdots & q_{1} & 1 \end{array}\right),\;0<q_{1}<1,\;q_{i+1}<q_{i},\;i=1,\ldots ,m-1$$

It can be shown that for h>0, the norm $\left\|Q_{1}\right\|=\underset{j}{\max}\left(\sum_{i}\left|\Theta_{i\;j}\right|\right)<\infty$.

System (17) can be represented in the following form:

(18) $$T=Q_{2}T+F$$

where

$$Q_{2}=\left(\begin{array}{cccccc} 0 & 0 & 0 & \cdots & 0 & 0\\ \;q_{1}^{*} & 0 & 0 & \cdots & 0 & 0\\ q_{2}^{*} & \;q_{1}^{*} & 0 & \cdots & 0 & 0\\ \vdots & \vdots & \vdots & \ddots & \vdots & \vdots \\ \;q_{m-1}^{*} & \;q_{m-2}^{*} & \;q_{m-3}^{*} & \cdots & \;q_{1}^{*} & 0 \end{array}\right),\;T=\left(\begin{array}{c} t_{1}^{\left(1\right)}\\ t_{1}^{\left(2\right)}\\ t_{1}^{\left(3\right)}\\ \vdots \\ t_{1}^{\left(m\right)} \end{array}\right),\;F=\frac{1}{c_{0}}\left(\begin{array}{c} \Phi_{1}\\ \Phi_{2}-\Phi_{1}\\ \Phi_{3}-\Phi_{2}\\ \vdots \\ \Phi_{m}-\Phi_{m-1} \end{array}\right)$$

$\;q_{1}^{*}=1-q_{1}$, $q_{i}^{*}=q_{i-1}-q_{i}$, $q_{i}^{*}>0$, i=2,…,m−1. Due to the fact that $\left\|Q_{2}\right\|=\sum_{i=1}^{m-1}q_{i}^{*}=$$1-q_{m}<1$ for h>0, the simple iteration routine [26,27] implies that the problem on solving the system of Equation (18), and, consequently, (17) is well-posed. Based on this fact, system (17) allows for deriving a recursive formula for determination of $t_{1}^{\left(l\right)}$, l=1,…,m.

Having derived the temperature $t_{1}(\tau )$, $\tau \in [0,\tau_{m}]$, by means of the foregoing routine, we can use Formula (14) to determine the temperature field within the tribo-couple. The thermal stresses and displacements can be computed accordingly by making use of the Formulae (11)–(13) and (15), along with the basic thermoelasticity equations [23].

## 4. Numerical Example and Discussion

In order to verify the proposed solution technique, consider a solution to the formulated identification problem for the tribo-couple, whose inner layer 1 is made of steel $(\lambda_{1}=21$ [Wt/(m × K)], $a{\;}_{1}=5.9\times 10^{-6}$ [m^2^/s], $\alpha {\;}_{T}^{\left(1\right)}=14\times 10^{-6}$ [1/K], $E_{1}=190$ [GPa], and $\nu_{1}=0.3$) and the outer one 2 is made of copper ($\lambda_{2}=381$ [Wt/(m × K)], $a{\;}_{2}=101.9\times 10^{-6}$ [m^2^/s], $\alpha {\;}_{T}^{\left(2\right)}=17\times 10^{-6}$ [1/K], $E_{2}=121$ [GPa], and $\nu_{2}=0.33$). 

Herein, we employ the following commonly used verification strategy [5] with two stages. In the first stage, we formulate a direct problem by imposing the temperature $t{\;}_{1}^{*}(\tau )$ on the inner circumference of the tribo-couple. Together with the given temperature on the outer surface and the interface thermal conditions (8), this would allow us to compute the thermal field in the tribo-couple. Making use of the determined temperature, a solution of the thermoelasticity problem (3)–(6) is constructed analytically. The latter solution can then be used to derive an expression for the circumferential strain on the outer surface of the tribo-couple. In the second stage, we formulate the inverse problem, where condition (10) is used together with the circumferential strain computed on the previous stage in order to restore the temperature on the inner surface by making use of the proposed algorithm. By comparing the solution of the inverse problem with the temperature $t{\;}_{1}^{*}(\tau )$ imposed when formulating the direct problem on stage 1, we can draw a conclusion about the efficiency of the algorithm. When solving the inverse problem in this stage, we also introduce some random small errors in the distribution of the circumferential strain in order to verify the stability of the algorithm. 

By following this strategy, let us first consider the direct heat-conduction and thermoelasticity problems by imposing the following boundary temperatures

(19) $$t{\;}_{1}^{*}(\tau )=T_{0}+B(1-\cos2\tau ),\;t{\;}_{2}^{*}(\tau )=T_{0}$$

and pressures $p_{1}(\tau )=CH(\tau )$ and $p_{2}(\tau )=0$, where B,C=const, H(τ) is the Heaviside step function, to determine the circumferential strain distribution on the outer surface $\rho =k_{2}$. Then, we can approximate the constructed strain within certain accuracy and use it as the input data for the inverse problem to determine the temperature $t{\;}_{1}^{\left(l\right)}$, l=1,…,m, on the inner surface $\rho =k_{1}$. By comparing the computed values $t{\;}_{1}^{\left(l\right)}$, l=1,…,m, with the actual $t_{1}(\tau )$, $\tau \in [0,\tau_{m}]$, imposed in (19), we can evaluate the accuracy of the proposed solution algorithm for the considered inverse problem of thermoelasticity. 

**Figure 2:**
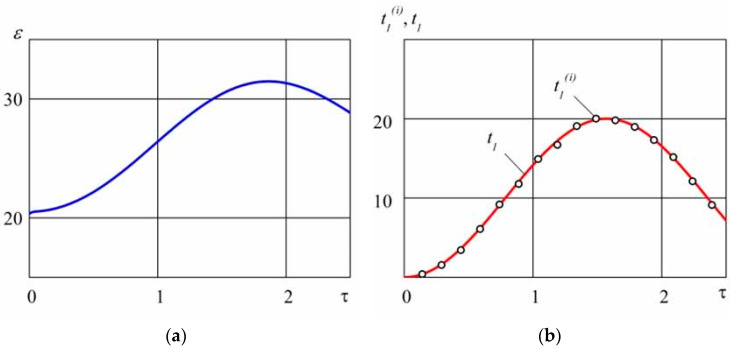
The circumferential elastic strain $\varepsilon (\tau )=\varepsilon_{\varphi \varphi }^{\left(2\right)}(k_{2},\tau )\times 10^{4}$ (**a**) computed by the temperature $t_{1}(\tau )$ given in (19) by solving the direct problem versus the dimensionless time τ; the dimensionless temperature $t{\;}_{1}^{\left(i\right)}$ on the inner circumference (**b**) as given by formula (19) (solid lines) and computed by solving the inverse problem (open circles).

The distribution of the dimensionless circumferential strain $\varepsilon (\tau )=\varepsilon_{\varphi \varphi }^{\left(2\right)}(k_{2},\tau )\times 10^{4}$ on the outer surface $\rho =k_{2}$ is shown in Figure 2a. The strain was computed from the direct problem under the thermal loading (19) for the following parameters $R{\;}_{0}=5.0\times 10^{-2}$ [m], $R{\;}_{1}=3.5\times 10^{-2}$ [m], $R{\;}_{2}=6.0\times 10^{-2}$ [m], $n{\;}_{1}=10^{-3}$ [m/GPa], $\;n_{2}=10^{-4}$ [m/GPa], $R=5.0\times 10^{-3}$ [m^2^ × K/Wt], $R{\;}_{*}=1.1\times 10^{-3}$ [m^2^ × K/Wt]; $\sigma_{*}=10^{2}$ [MPa], $T_{0}=20$ [K], B =200 [K], $C=10^{2}$, f=0.25, ω=1.22 [rad/s], and $\tau {\;}_{m}=2.5$. 

Now we can use the computed strain as the input data for solving the inverse problem in order to reconstruct the thermal loading on the inner circumference of cylinder 1. It is also important to analyze the effect of small errors in the input data (which can be induced by the errors in the stain measurement, etc.). For modeling of such errors, let us substitute the strain distribution at the discrete time moments $\tau_{i}$ with the values $\tilde{\varepsilon }(\tau_{i})$ computed by the formula $\tilde{\varepsilon }(\tau_{i})=\varepsilon (\tau_{i})(1+\theta_{i}\times 10^{-2})$, where $\theta_{i}$ are arbitrary numbers from the interval [−1, 1] with the uniform distribution law and represent $\tilde{\varepsilon }(\tau )$ by a linear spline. This means that the input data are encountered with an arbitrary error falling within 1%. 

In Figure 2b, the open circles denote the time distribution of the temperature $t{\;}_{1}^{\left(i\right)}$, $i=\bar{1,250}$ on the inner surface of the cylinder 1, found by solving the inverse thermoelasiticy problem with the computational step h=0.01. It is shown that the maximum relative error of the computed values in comparison to the corresponding values imposed in the direct problem (19) falls within 1.8%, which confirms the stability of the proposed solution algorithm with respect to the small errors in the input data. Due to the fact that the solutions to well-posed direct problems are stable with respect to small errors in the input data, the error in computing the thermal stresses, strains, and displacements by using the thermal loading (19) of the one computed by solving the inverse problem can be dismissed.

## 5. Conclusions

A problem on the determination of temperature and thermal-stress fields in a cylindrical tribo-couple with frictional heating on the interface is formulated for the case when the thermal loading on one of its circumferences is unknown. The additional information about the transient variation of the circumferential strain on the surface where the thermal loading is known was used as a supplementary condition for the formulated inverse thermoelasticity problem governed by a Volterra integral equation of the first kind. Due to the fact that the kernel of this integral equation $K_{1}(\tau -\eta )$ takes only positive values on the interval η∈[0,τ], monotonically increases for the entire range of variables, and suffers a root singularity at the point η=τ, this integral equation can be regarded as one of Abel kind. The presence of the integrable singularity in this kernel at η=τ implies that there is no delay in the maximum thermal response of the circumferential strain $\varphi_{*}(\tau )$ to a change in the variation profile of temperature $t_{1}(\tau )$ at η=τ in view of the integral dependence of this strain on the temperature within the cylindrical layers of the tribo-couple. This feature of kernel $K_{1}(\tau -\eta )$ ensures the conditional correctness of the inverse problem. The correctness condition in this case was derived in the form of the fitting condition for the, circumferential strain on the periphery of the tribo-couple, and the pressures applied to its surfaces at the initial moment of time.

It is worth noting that the analogous kernels within the framework of inverse heat-conduction problems solely exhibit quite different features, which, in the final count, makes these problems ill-posed [5,6].

Another advantage of the proposed technique is that the system of algebraic Equation (17), which is the discrete analog for Equation (16), was represented in the form (18). This, in view of the appearance of its matrix $Q_{2}$ ensures the stability of its solution with respect to small errors in the input data. An algorithm for solving the formulated inverse problem is suggested on the basis of the linear spline approximation technique. The efficiency of the algorithm was verified by solving the direct problem under the given thermal loading in order to determine the circumferential strain, which was then used as the input data for the inverse problem on the reconstruction of thermal loading. 

These key features of the proposed algorithm may serve for benefit of setting up technological and experimental cylindrical tribo-systems expecting incomplete information about thermal loading for engineering applications and the wear analysis [28,29].

## Data Availability

Data Sharing is not applicable.
